# Experimental Research on Vibration-Damping Effect of Combined Shear Hinge Prefabricated Steel Spring Floating Slab Track

**DOI:** 10.3390/s22072567

**Published:** 2022-03-27

**Authors:** Zhiping Zeng, Xudong Huang, Zhuang Li, Weidong Wang, Zixiao Shi, Yu Yuan, Abdulmumin Ahmed Shuaibu

**Affiliations:** 1 School of Civil Engineering, Central South University, Changsha 410075, China; 203160@csu.edu.cn (Z.Z.); zhuang_li2@yeah.net (Z.L.); wd1997@csu.edu.cn (W.W.); 8210200726@csu.edu.cn (Z.S.); abdulshub4u@csu.edu.cn (A.A.S.); 2 MOE Key Laboratory of Engineering Structures of Heavy Haul Railway, Central South University, Changsha 410075, China; 3 Information Engineering School, Nanchang University, Nanchang 330031, China; yuyuan@email.ncu.edu.cn; 4 Department of Civil Engineering, Faculty of Engineering, Ahmadu Bello University Zaria, Kaduna 800242, Nigeria

**Keywords:** subway, combined shear hinge, steel spring floating slab track, prefabricated, vibration isolator

## Abstract

Objective: The cast-in-place steel spring floating slab track (SSFST) is difficult to maintain and repair, while the mechanical strength of the end of the traditional prefabricated SSFST is poor. In order to overcome the above shortcomings, a shear-hinge-combined prefabricated SSFST was developed, and an indoor test was carried out to analyze its vibration-damping effect. Methods: A combined shear hinge SSFST connection model with two length sizes was established. The dynamic response amplitude and frequency response characteristics of the foundation (ground) under different isolator installations and fatigue loads were studied, and the vibration-damping performance of two sizes of combined shear hinge SSFST was evaluated. Results: The vibration-damping effect of the steel spring vibration isolator mainly acts in the middle and low-frequency bands of 16–400 Hz, and the vibration near 10 Hz will be aggravated after the vibration isolator is installed. The vibration index and variation law of the two sizes of SSFST are similar, and the vibration response of 4.8 m SSFST is slightly less than 3.6 m SSFST. There is almost no change in each index when the load is 5 million times, and there is a certain range of change when the load is 10 million times, but the overall change is small. Conclusions: The combined shear hinge prefabricated SSFST can have an excellent isolation effect on vibration and can still maintain good vibration-damping ability within 10 million fatigue loads (about 5 years); 4.8 m SSFST should be laid in straight sections with higher train speeds, while 3.6 m SSFST should be applied in curved sections to ensure smooth lines.

## 1. Introduction

With the rapid development of the rail transit industry worldwide and the further acceleration of urbanization, most economically developed cities need an urban railway system between the main railway and the general low-speed urban rail transit to further provide linkage between urban and suburban areas [1,2,3]. Therefore, urban express rail transits have been rapidly developed to meet the medium- and long-distance travel requirements of urban residents [4,5]. However, due to the normally dense land use in urban areas, urban express rail transits often pass very close residential areas [6,7,8]. For this reason, urban express rail transit is likely to have a greater impact on the environment, particularly in terms of vibration and noise caused by train operation. Therefore, there is a need for an efficient vibration damping measure to solve this increasingly serious problem [9].

Among all urban rail transit vibration isolation measures, the floating slab track (FST) is one of the most efficient vibration-damping structures and has some outstanding advantages [10,11]. In comparison with an integrated slab track system, the FST more effectively reduces the transmission of force [12,13]. However, steel spring FST (SSFST) represents one of the best methods of urban rail transit damping currently recognized and has been widely used around the world [14]. For example, the Japan Tsukuba Express Line, built in 2005, has an SSFST section that has facilitated a maximum speed of 160 km/h. However, South Korea Busan and Cheonan Stations also adopted SSFST for vibration damping and subsequently recorded speeds of 350 km/h, making them the world’s fastest and most heavily loaded FST application case [15].

As the speed of the train increases, the unevenness when the train passes the end of the slab is reduced, the force on the end of the track slab of the steel spring floating slab of different lengths is improved, and the shear hinge device needs to be arranged between the track slabs [16]. However, whether the shear hinge will affect the vibration-damping effect of the floating slab track under the train impact, and whether the shear hinge will affect the vibration of the two types of floating slab track under different fatigue loads, are questions that need to be investigated. Therefore, it is necessary to analyze the vibration-damping performance of the floating slab track under the combination of shear hinges, and to establish a full-scale model of the prefabricated steel spring floating slab track with two lengths of shear hinge combination, which could be used to analyze the dynamic response amplitude and frequency response characteristics of the track foundation under different fatigue loads.

In the process of engineering practice, the vibration-damping effect of SSFST is particularly valued by the owner and design unit, and there are few studies on the vibration-damping performance of the prefabricated steel spring track system under fatigue load. Therefore, in order to analyze the vibration-damping performance of SSFST after fatigue load, the ground (foundation) measuring point 150 cm from the longitudinal centerline of SSFST (that is, 15 cm from the edge of the slab) is taken as the research object. Through indoor fatigue tests on SSFST with two lengths of 3.6 m and 4.8 m, the time domain, frequency domain, and vibration level characteristics of the foundation vibration when SSFST is subjected to different times of fatigue loads are analyzed, which could provide a theoretical reference for related engineering applications.

Therefore, two full-scale combined shear hinge prefabricated SSFST models with different lengths are established to analyze the dynamic response amplitude and frequency response characteristics at the track foundation under different fatigue loads and different impact positions. The vibration law and frequency response distribution characteristics at the foundation of two kinds of combined shear hinge SSFST structures are explored, and the vibration-damping performance of two kinds of combined shear hinge SSFST structures is evaluated, which accumulates effective test data for the study of vibration-damping performance of combined shear hinge SSFST.

## 2. Model Building and Test Plan

### 2.1. Full-Scale Model of SSFST

In order to ensure the safe and stable operation of trains and save the number of fasteners, the distance between fasteners in Chinese subways is generally set to 0.6 m [17]. If the steel spring vibration isolator is to be set in the design, at least 2 rows of fasteners are required, then the track slab needs to be set to an integer multiple of 1.2 m, that is, the lengths of 1.2 m, 2.4 m, 3.6 m, 4.8 m, 6 m, etc. When the length of the track slab is too short, the structural vibration is unstable and is not conducive to the safety of the train. If the length of the track slab is too long, it is not conducive to construction and hoisting and it is difficult to lay in the curved section. According to the theoretical calculation of relevant research results, it is found that the dynamic indicators decrease with the increase of the track slab length. When the track slab length exceeds 3.6 m, the reduction degree of the dynamic effect will no longer be obvious. Therefore, the lengths of 3.6 m and 4.8 m are selected in the design.

According to the prefabricated SSFST type laid in the subway main line tunnel, two track bed slabs (3.6 m and 4.8 m) were selected for the drop hammer impact test, and the vibration acceleration of the ground (foundation) measuring point 15 cm away from the edge of the SSFST slab was tested. The SSFST structure is mainly composed of steel rails, spring bars VII type subway fasteners, track bed slabs, vibration isolators, and shear hinges.

**Figure 1:**
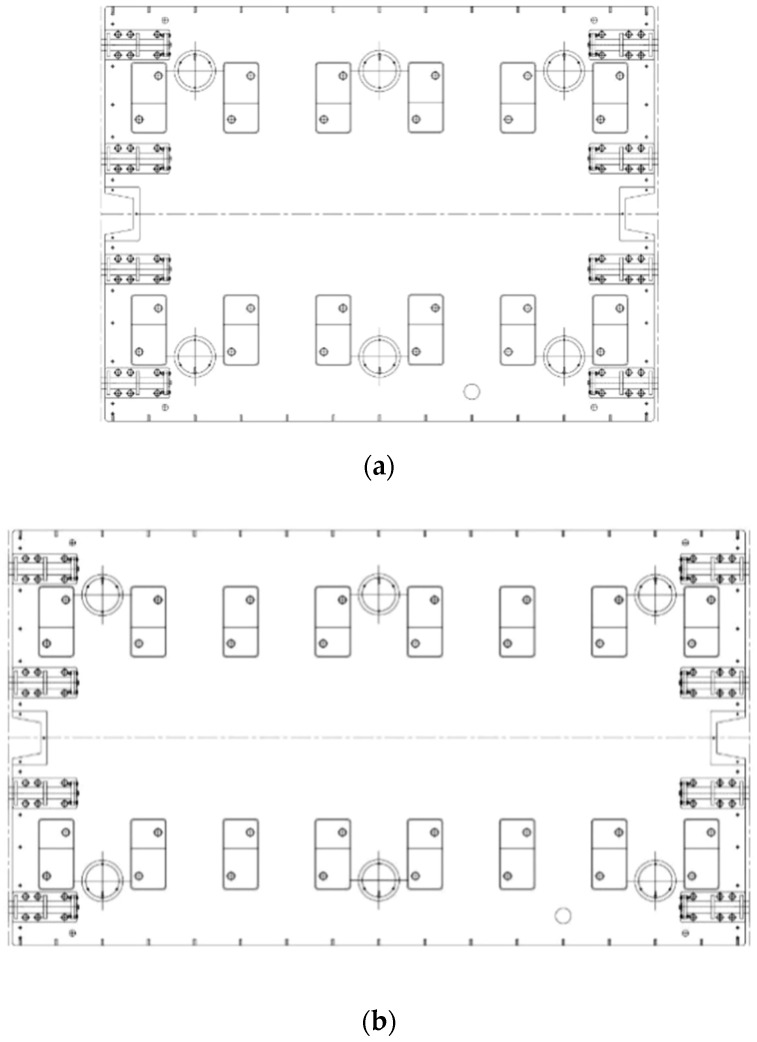
Schematic diagram of floating slab track. (**a**) Design drawing of 3.6 m SSFST. (**b**) Design drawing of 4.8 m SSFST.

The prefabricated SSFST adopts a 3-3 vibration isolator arrangement and consists of a complete assembly structure consisting of steel springs, damping, leveling washers, locking slabs, locking bolts, upper shear hinges, and prefabricated slabs, as shown in Figure 1. SSFST test component parameters are shown in Table 1.

**Figure 2:**
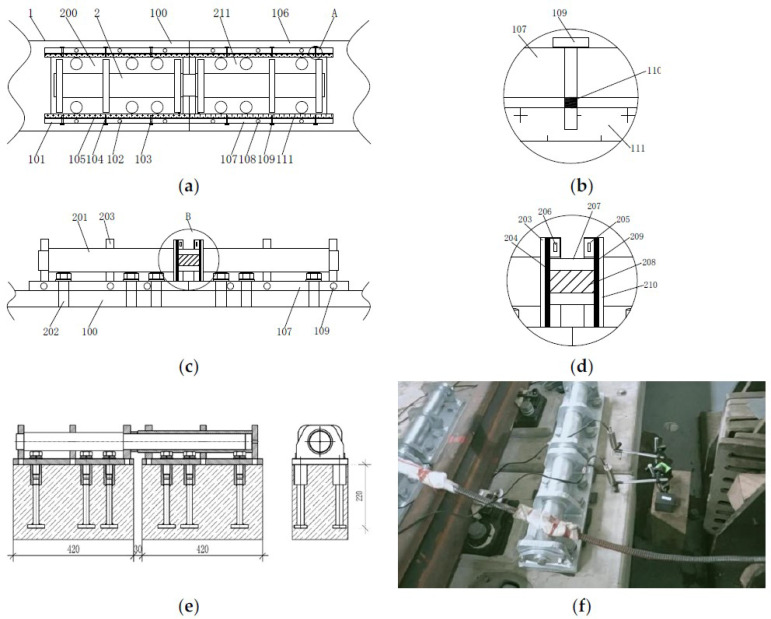
Floating slab track combined shear hinge structure design and physical site drawing. (**a**) Top view schematic. (**b**) The enlarged schematic diagram at A. (**c**) Side view schematic. (**d**) The enlarged schematic diagram at B. (**e**) Design drawing of shear hinge structure. (**f**) On-site installation of shear hinge.

The design schematic of the combined shear hinge and the site are shown in Figure 2. When the shear hinge device is in use, the first shear hinge clamping plate 105 and the second shear hinge clamping plate 111 are moved to the top of the first floating slab 100 by the first adjusting bolt 103 and the second adjusting bolt 109. The shear hinge 201 is placed in front of the first floating slab 100 and the second floating slab 106, and the first shear hinge base 200 is threadedly connected to the first floating slab 100 through fixing bolts 202. The first adjusting bolt 103 is adjusted to make the first shear hinge clamping plate 105 contact and clamp the first shear hinge base 200, which effectively prevents the shear hinge 201 from breaking due to loosening of the fixing bolts 202 in front of the first shear hinge base 200. The movable baffle 205 is taken out through the baffle connecting block 206, and the first connecting plate 204 and the second connecting plate 209 are put into the interior of the first shear hinge support 203 and the second shear hinge support 210, where the damping sleeve 207 and the spring 208 can achieve a certain buffering effect. After the placement is completed, the movable baffle 205 is reset, so that the staff can replace the damping sleeve 207 and the shear hinge 201 at any time. The second shear hinge base 211 is fixed to the front of the second floating slab 106 by the fixing bolts 202, the second adjusting bolts 109 are adjusted; when the second shear hinge clamping plate 111 is brought into contact with the second shear hinge base 211 and clamped, the installation work of the device can be completed [18].

### 2.2. Drop Hammer Impact Test Instrument

The drop hammer impact test is to test the vibration acceleration of the SSFST foundation under the drop hammer impact before and after the installation of the spring isolator, after 5 million times and 10 million times of fatigue tests. It is possible to understand the vertical vibration acceleration, frequency spectrum characteristics, and Z vibration level of the SSFST and its foundation under the impact of the drop weight before the installation of the spring isolator, after the installation and after the fatigue test, and to grasp the vibration-damping effect of the spring SSFST vibration isolator after the installation of the spring SSFST vibration isolator under the impact of the falling weight, and after the fatigue test of 5 million times and 10 million times, relative to the vibration isolator before the installation of the vibration isolator.

**Figure 3:**
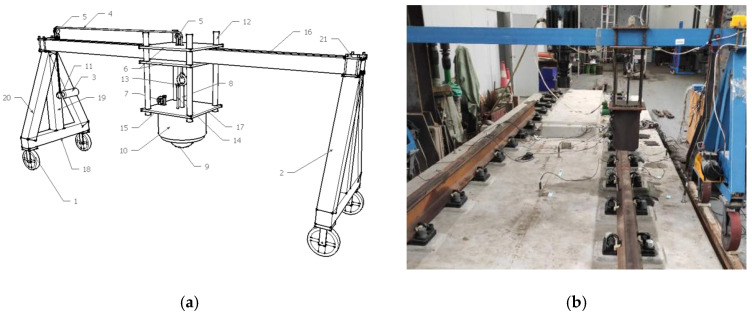
Drop hammer impact test device. (**a**) Design drawing. (**b**) Site installation.

The drop hammer impact device used in the test can accurately position the drop hammer at the required height and accurately control the position of the hammer body impacting the surface of the rail. The drop weight frame can move freely on the beam and can also provide different drop weight heights, which is suitable for the drop hammer impact test of different sizes and different types of tracks, as shown in Figure 3.

Among them, the weight of the drop hammer is 50 kg, the vertical drop height is 100 mm, and the hammering points at the two rails of the SSFST (the hammering points are located at 1/2 of each of the two sizes of SSFST) and no fewer than 10 effective impacts are performed.

### 2.3. Fatigue Load Test System

The type of fatigue machine used in this test is PMW800-500 electro-hydraulic type, and its hydraulic pulse displacement is 800 mL/min. The whole device includes the host and controller. The hydraulic head can apply a maximum pressure of 500 kN. Combined with the gantry loading device and special railway auxiliary appliances, up to four hydraulic actuators can be configured for loading at the same time. Therefore, the test system is mainly composed of SSFST system, loading device, data acquisition device, and so on.

**Figure 4:**
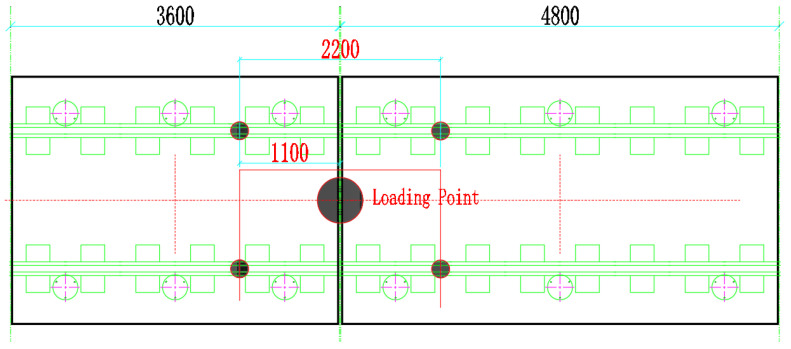
Schematic diagram of fatigue test system of SSFST system model.

**Figure 5:**
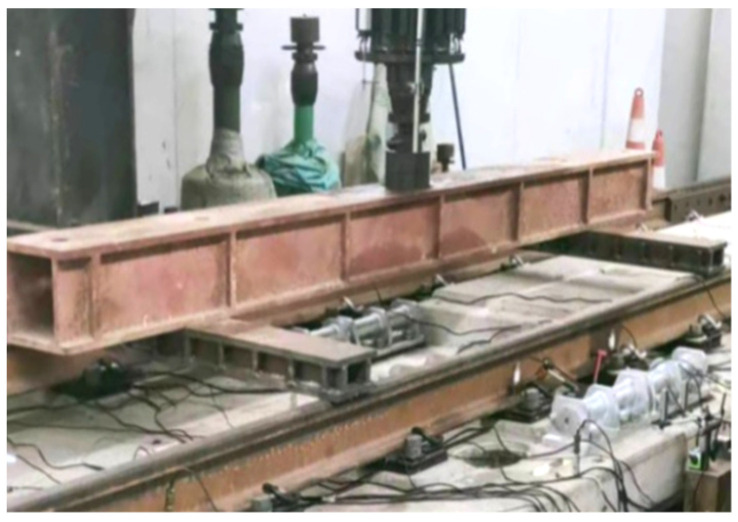
Loading device site.

The schematic diagram of the test system is shown in Figure 4, and the field of the loading device is shown in Figure 5. During the fatigue load test, the rail is pressed by the force adding frame. Load value 80 kN < P < 308 kN, number of cycles N1 = 5 × 10^6^, N2 = 1 × 10^7^, and the load frequency is 3 Hz. The reason is that the axle load of Chinese subway type B car is 14 t [19], and one bogie has two axles (weight is 28 t), and then multiplied by the safety factor of 1.1, so the upper limit of the fatigue load is taken as 28 × 10 × 1.1 = 308 kN. The lower limit of fatigue load = upper limit × 0.25, so on the basis of 308 kN, 308 × 0.25 = 77 kN, taking a multiple of 10 kN is 80 kN. The length of Chinese subway type B car is 12.6 m, each car has 2 bogies, and the actual running speed of the train is generally 70 km/h (19.44 m/s), so in the average time interval of 12.6/(2 × 19.44) = 0.324 s, the track slab will pass a bogie load, which is about 3 Hz. Therefore, the fatigue load value in this study can simulate the real train load well and has reliability.

### 2.4. Introduction to the Test Plan

#### 2.4.1. Test Point Layout

**Figure 6:**
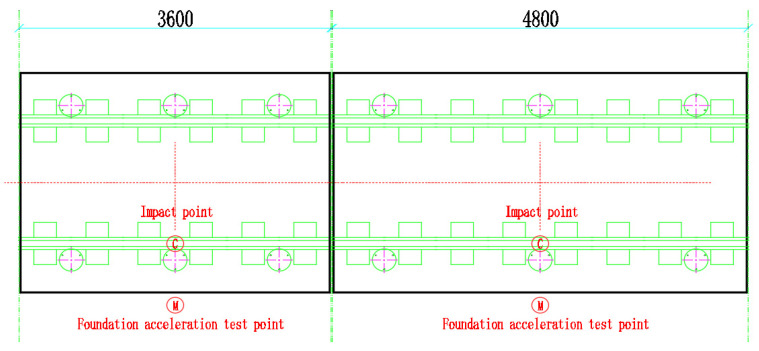
Layout of the accelerometer measuring points.

Since the function of the shear hinge is the same under different settings, it provides the establishment of longitudinal shear force constraints for the end of the track slab, and the end conditions of different track slabs are similar. In order to study the vibration-damping performance of the track slabs with two lengths at the same time, under the same experimental cost and conditions, 3.6 m + 4.8 m is the best experimental plan. To fully explore the damping performance of SSFST, the acceleration at the ground foundation is mainly collected. Therefore, the acceleration sensors are arranged on the foundation of the track slab, and the frequency response characteristics of the foundation under different working conditions are explored through the comparative analysis of the acceleration at the foundation of each track. In the test, the drop hammer impact test is mainly performed on two types of track slabs. When arranging the acceleration measuring points, the 0.5 g acceleration sensor is mainly arranged on the ground in the middle of the SSFST, as shown in Figure 6.

#### 2.4.2. Test Sampling Parameters and Data Processing Principles

In order to collect more accurate data, the collection frequency of each parameter is set to 20,000 Hz. When organizing test data, system errors should be eliminated, and suspicious data generated by negligence errors should be discarded. The time-domain waveform should be pre-checked to remove singular items, correct zero-line drift, remove trend items, and remove other errors to ensure the accuracy and authenticity of data analysis [20].

According to the setting of the test conditions, in order to explore the influence of the falling weight impact on the vibration damping characteristics of SSFST, the analysis is carried out from the time domain and frequency domain, respectively. A comparative study is undertaken of the basic vibration characteristics of SSFST without vibration isolator, with vibration isolator, after 5 million fatigue cycles, and after 10 million fatigue cycles, combining the impact and vibration attenuation analysis results of the two SSFST structures and the vibration conditions of the foundation under different fatigue times, and then the vibration-damping performance of the two subway SSFST structures is evaluated.

## 3. Time Domain and Frequency Domain Analysis

### 3.1. Time Domain Analysis

The purpose of the test research is mainly to study the vibration-damping performance of the two SSFST structures. Since the main purpose of SSFST is to reduce the vibration of the foundation, it is mainly concerned with the vertical acceleration response of the vibration at the foundation under the impact load of the falling weight.

Due to the environmental vibration around the laboratory during the test, a certain amount of clutter will be generated, and the accelerometer at the foundation has high accuracy and is very susceptible to the influence of small external vibrations. Therefore, in order to eliminate the influence of clutter on the test analysis, it is necessary to reduce the noise of the collected signal.

Among the current denoising methods, wavelet threshold denoising has a better application [20,21,22]. The basic method is to use a family of functions to approximate the objective function hierarchically. Among them, the generated function *Ψ*(*t*) will to meet the laws (1) and (2):

Time domain:


(1) $$\int_{-\infty }^{\infty }\Psi (t)dt=0$$

Frequency domain:

(2) $$C_{\psi }=\int_{-\infty }^{\infty }\frac{\left|\varphi^{*}(w)\right|}{\left|w\right|}dw<\infty$$

where *φ*(*w*) is the Fourier transform of *Ψ*(*t*) and φ*(*w*) is the complex conjugate function of *φ*(*w*).

When selecting the basis function and decomposition series of the vibration acceleration signal at the basis of this research, the following strategies are adopted:

(1) Intercept the original signal for 0.4 s, use wavelet soft threshold denoising decomposition to denoise, and use the default threshold for the threshold. A three-level wavelet decomposition is performed on the basic vibration signal, and an approximation signal is obtained.
(2) Reconstruct the decomposed signal to obtain the noise-reduced basic vibration signal for subsequent frequency domain and vibration level analysis.

The dbN wavelet denoising method is adopted in this study (db is the abbreviation of Daubechies, and N is the wavelet order). The number of wavelet decomposition layers is one of the factors affecting the effect of wavelet noise reduction. The larger the number of decomposition layers, the more obvious the difference between noise characteristics and signal characteristics will be, which is more favorable for signal and noise separation. As far as reconstruction is concerned, the larger the number of decomposition layers, the larger the error in reconstruction and the greater the distortion; when the number of decomposition scales is too small, the maximum value of the modulus corresponding to the noise cannot be sufficiently attenuated, which will cause the signal to be indistinguishable from the noise. It can be seen that if the decomposition scale is too large, some important local features in the signal will be lost. Therefore, it is often decomposed into 3~6 levels when performing wavelet noise reduction. In this study, the signal-to-noise ratio has reached 70.7013 when it is decomposed into three levels, indicating that the noise signal is greatly reduced. In order to ensure that important local features in the signal are not lost, level 1, 2, and 3 wavelet decomposition are selected [23].

The mathematical characteristics and smoothness of the signal after soft threshold processing are also better, and the processing result is also more reliable. Therefore, we choose the dbN wavelet denoising method with soft threshold. In order to ensure that the signal is not distorted after noise reduction, we used the corresponding multi-scale one-dimensional wavelet decomposition function “wavedec” when using MATLAB to calculate, and set up a three-level decomposition function to achieve the removal of high-frequency white noise [24].

**Figure 7:**
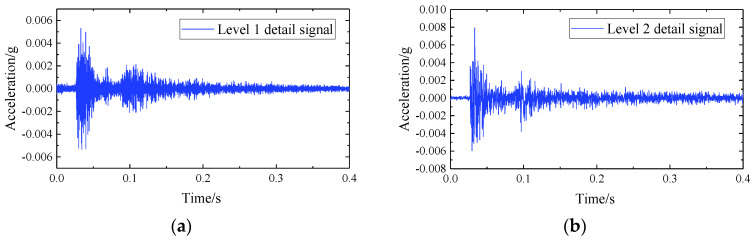

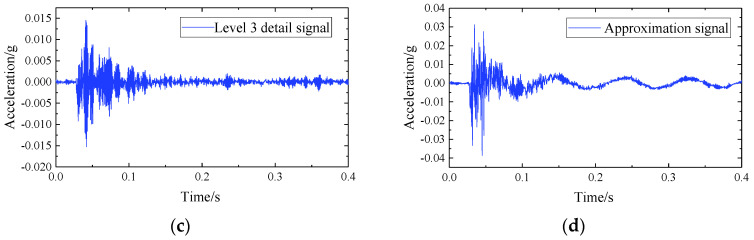
Wavelet soft threshold de-noising decomposes the detailed signal. (**a**) Level 1 detail signal. (**b**) Level 2 detail signal. (**c**) Level 3 detail signal. (**d**) Approximation signal.

**Figure 8:**
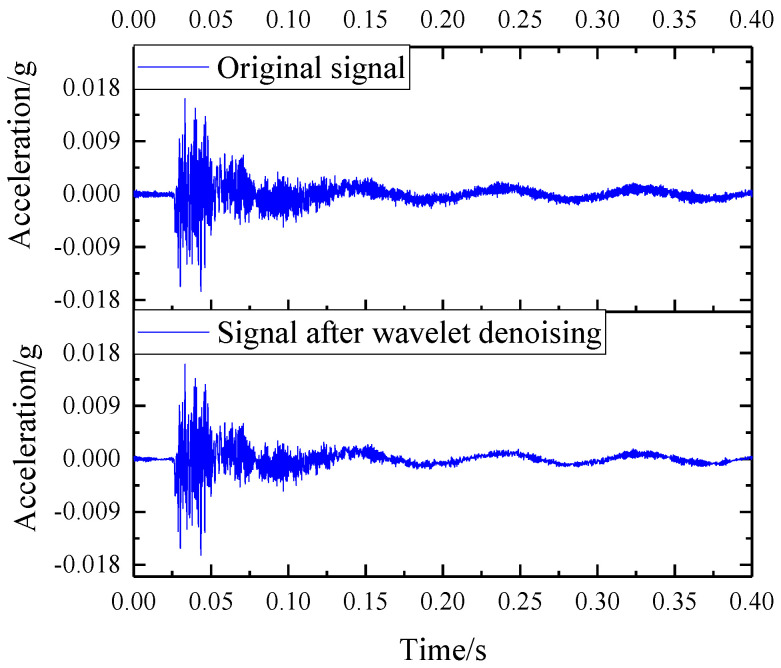
Comparison of the original signal and the signal after wavelet denoising.

Take the acceleration data of the measuring point at the foundation after the vibration isolator is installed at 3.6 m SSFST as an example, as shown in Figure 7. After reconstructing the decomposed signal, it can be seen that the overall waveform of the signal after denoising has not changed much, but the clutter of the signal has been significantly reduced; as shown in Figure 8, 0~0.03 s and 0.17~0.30 s are the most significant, indicating that the wavelet soft threshold denoising is suitable for this type of signal processing. After clutter processing, it will be more conducive to subsequent analysis.

**Figure 9:**
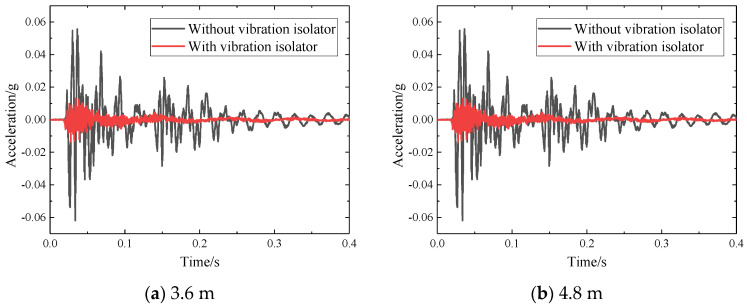
Acceleration time history waveform with or without vibration isolation measures.

**Figure 10:**
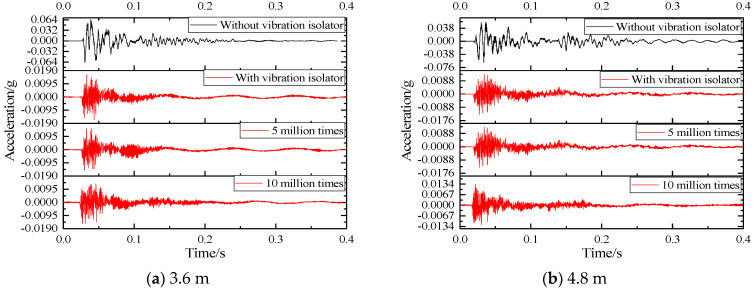
Basic acceleration time history waveform for different fatigue times.

The change law of the acceleration time history waveform of the track structure components at the basic acceleration measurement point is relatively similar. Therefore, for different parts of the basic measuring point, the measuring point closest to the impact point is selected for analysis, which is 15 cm away from the edge of the slab. The time-history waveforms of vibration acceleration of two SSFST structural components are shown in Figure 9 and Figure 10.

According to Figure 9 and Figure 10:

(1) During the SSFST drop hammer impact test, it can be seen that the positive value of acceleration at the foundation is slightly larger than its negative value. After the vibration isolator is installed, its peak acceleration is greatly reduced, and its drop is about 80%, which shows that the vibration isolator has a key effect on reducing the dynamic response of the SSFST system to the foundation.
(2) After the prefabricated SSFST is installed with the vibration isolator, the period of shock and vibration level is continuously shortened. After the installation of the vibration isolator, the total vibration duration of the SSFST is less than the prefabricated SSFST without the vibration isolator. It can be seen that the SSFST with the vibration isolator can achieve excellent vibration energy absorption.
(3) SSFST of two lengths has similar basic vibration laws, and both acceleration amplitude and attenuation waveform are similar. Among them, the acceleration of 4.8 m SSFST is slightly smaller than 3.6 m SSFST, which indicates that the length of the track slab has a small effect on the attenuation of the foundation vibration.
(4) With the application of fatigue load, the waveform of the acceleration waveform at the foundation has slight changes, but the overall change is small.

The time domain analysis mainly focuses on the peak value, peak-to-peak value, and effective value of the track structure impact vibration. The specific meaning is as follows:

(1) The peak value mainly represents the maximum value of the acceleration at the foundation when the SSFST is subjected to an impact, which can directly reflect the acceleration of the foundation when the impact is received.
(2) The peak-to-peak value is the difference between the maximum value and the minimum value of the acceleration at the foundation, and its magnitude has a greater correlation with the shock received at the foundation, so it can be used as an important indicator for evaluating the shock response received at the foundation.
(3) The effective value is also called root-mean-square value, which can represent the vibration energy generated when the foundation is impacted.

Table 2 shows the peak and effective values of vibration acceleration of the two SSFST foundations under different assembly conditions and fatigue loading times of the vibration isolator.

**Figure 11:**
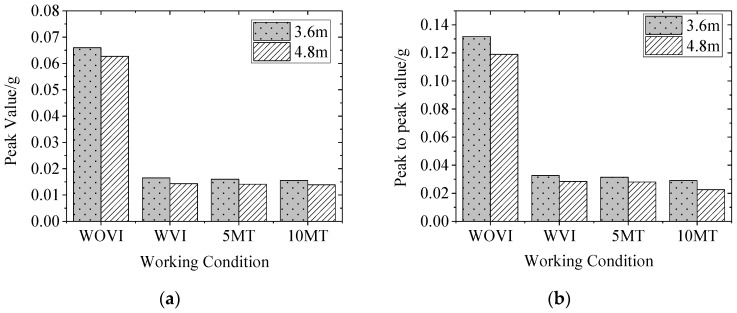

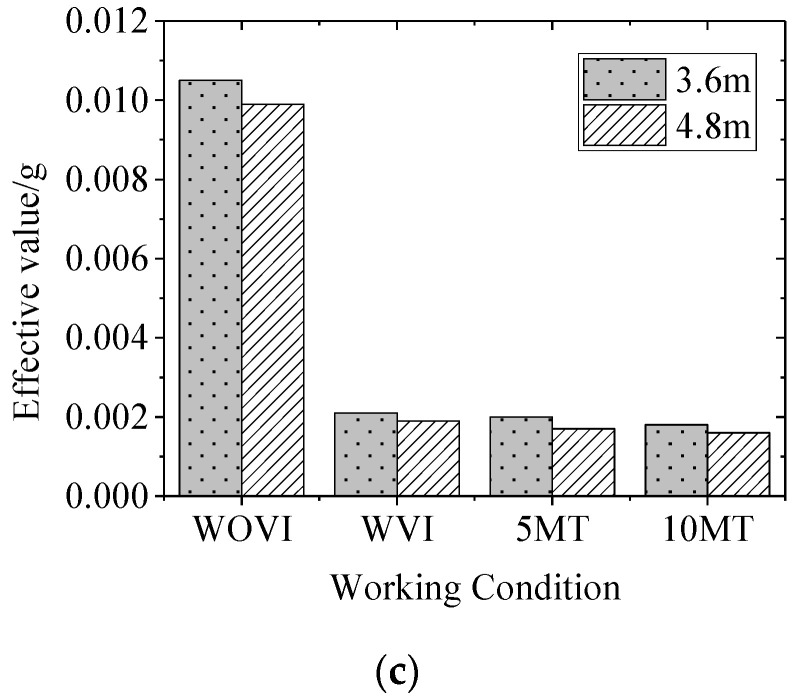
Comparison diagram of time-domain analysis of vibration acceleration. (**a**) Peak value. (**b**) Peak to peak value. (**c**) Effective value.

Among them, the effective value is calculated by selecting the entire vibration period, and the duration is set to 0.40 s and is shown in Figure 11; it can be seen that:

(1) After the installation of the vibration isolator, the foundation acceleration peak, peak-to-peak value, and effective value are significantly reduced, and the drop rate is about 80%. Therefore, SSFST is beneficial to reduce the vibration response level of the track structure.
(2) When the number of fatigue loads increases, the maximum, minimum, peak, and effective values of the two SSFST foundations have little change, and the overall decrease slightly. It can be seen that the vibration effect of this type of SSFST is less affected by the fatigue load.
(3) Comparing the peak and effective value of vibration acceleration at the foundation under different assembly conditions of vibration isolators and the number of fatigue loads, it can be seen that the vibration response amplitude of 3.6 m SSFST is slightly larger than 4.8 m SSFST, and the acceleration level of both at the foundation is generally low.
(4) When laying SSFST tracks in subway tunnels, when wheel-rail impact occurs, it is beneficial to reduce the vibration response of the tunnel base and lining structure and reduce the possibility of damage to the track and tunnel structure.

### 3.2. Frequency Domain Analysis

In the frequency domain analysis of vibration signals, frequency spectrum is often used as an important research method. Through the transformation of parameters such as amplitude in different frequency ranges, the amplitude and phase contained in the vibration signal can be displayed. In the analysis, the Fourier transform is the most used. When in use, the frequency and time domain of the vibration signal can be related to each other, and then the signal characteristics contained in the time domain signal can be displayed and used for analysis. Therefore, the Fourier transform has been widely used [25,26].

In this study, based on the FFT method, the vibration signals at the foundation of the prefabricated SSFST under different fatigue times were transformed. Since the amplitude spectrum (RMS) mainly reflects the effective value of the vibration acceleration signal, it has a good reflection on the distribution of the vibration signal in each frequency band. Therefore, a comparative study was made on the amplitude spectrum (RMS) of the foundation shock vibration.

**Figure 12:**
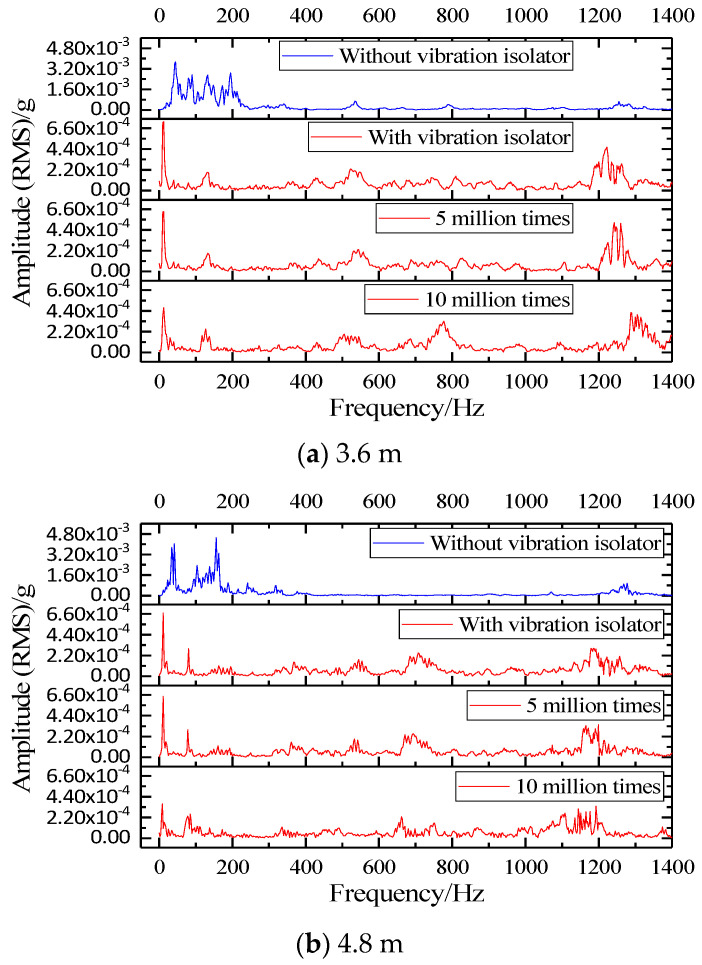
SSFST basic amplitude (RMS) comparison waveform.

In order to study the vibration distribution of the foundation in different frequency domains under different working conditions, the amplitude spectrum (RMS) of the foundation is obtained through fast Fourier transform (FFT), as shown in Figure 12. The amplitude spectrum (RMS) can well show the changing law of the amplitude waveform under different working conditions, that is, it is easier to compare and analyze.

According to Figure 12a, when the vibration isolator is not installed, the amplitude spectrum (RMS) of 3.6 m SSFST has 7 obvious peak points, located at 44.99 Hz, 89.98 Hz, 132.48 Hz, 194.98 Hz, 534.93 Hz, 789.91 Hz, and 1254.84 Hz, and their RMS is 37.28 × 10^−4^ g, 27.27 × 10^−^^4^ g, 27.12 × 10^−4^ g, 28.73 × 10^−4^ g, 6.79 × 10^−4^ g, 4.19 × 10^−4^ g, and 6.54 × 10^−4^ g, respectively. After installing the vibration isolator, the amplitude spectrum (RMS) has four obvious peak points, located at 10.50 Hz, 134.98 Hz, 524.93 Hz, and 1222.35 Hz, its RMS is 7.31 × 10^−4^ g, 1.91 × 10^−4^ g, 2.23 × 10^−4^ g, and 6.49 × 10^−4^ g, respectively. After 5 million fatigue loads, the positions and amplitudes of the above four peak points have relatively small changes, which are located at 10.25 Hz, 132.48 Hz, 542.43 Hz, and 1243.34 Hz, respectively, and its RMS is 6.34 × 10^−4^ g, 1.91 × 10^−4^ g, 2.30 × 10^−4^ g, and 5.16 × 10^−4^ g. The peak point and amplitude of 10 million times of fatigue change greatly, and another peak point appears at 777.41 Hz; all peak points are located at 10.498 Hz, 127.42 Hz, 504.43 Hz, 777.41 Hz, and 1287.34 Hz, respectively, and its RMS is 4.74 × 10^−4^ g, 2.49 × 10^−4^ g, 1.94 × 10^−4^ g, and 4.25 × 10^−4^ g, respectively.

The basic vibration acceleration of 4.8 m SSFST shown in Figure 12b also obeys a similar law, that is, the spectrogram changes greatly when the fatigue load is 10 million times. When the vibration isolator is not installed, the amplitude spectrum (RMS) of 4.8 m SSFST has 3 obvious peak points, located at 41.355 Hz, 156.23 Hz, and 1277.43 Hz, and its RMS is 40.46 × 10^−4^ g, 45.26 × 10^−4^ g, and 9.61 × 10^−4^ g, respectively. After installing the vibration isolator, there are 7 obvious peak points, located at 9.51 Hz, 80.41 Hz, 163.12 Hz, 367.61 Hz, 544.51 Hz, 707.63 Hz, and 1185.53 Hz, and the RMS is 3.67 × 10^−4^ g, 2.93 × 10^−4^ g, 1.11 × 10^−4^ g, 1.51 × 10^−4^ g, 1.76 × 10^−4^ g, 2.46 × 10^−4^ g, and 2.95 × 10^−4^ g, respectively. After fatigue loading, the positions of the above peak points have changed. After 5 million fatigue loads, the positions and amplitudes of the above seven peak points have relatively small changes, located at 9.49 Hz, 78.12 Hz, 160.82 Hz, 360.71 Hz, 533.02 Hz, 693.85 Hz, and 1164.85 Hz, and its RMS is 6.47 × 10^−4^ g, 2.92 × 10^−4^ g, 1.21 × 10^−4^ g, 1.60 × 10^−4^ g, 1.93 × 10^−4^ g, 2.47 × 10^−4^ g, and 3.34 × 10^−4^ g, respectively. The peak point and amplitude change greatly when fatigue is 10 million times. The number of obvious peak points is 6, located at 9.19 Hz, 85.01 Hz, 335.42 Hz, 661.68 Hz, 751.29 Hz, and 1192.42 Hz, and its RMS is 3.63 × 10^−4^ g, 2.56 × 10^−4^ g, 1.18 × 10^−4^ g, 2.27 × 10^−4^ g, 1.59 × 10^−4^ g, and 3.41 × 10^−4^ g, respectively.

## 4. Hilbert–Huang Transform Analysis

The shock component in the vibration signal of the SSFST system accounts for a large proportion, the frequency component is very rich, and the vibration information contained in different frequency bands is different [27]. When analyzing the vibration signal of the SSFST system, the Intrinsic Mode Function (IMF) components of each order after the Empirical mode decomposition (EMD) will contain the natural vibration components of different frequency bands caused by different vibration isolation methods [28,29,30,31]. According to the theory of kurtosis coefficient, IMF components with larger kurtosis values have more periodic impact components [32].

Due to the effect of the vibration isolator of the SSFST system, the dynamic response of the SSFST structure will have a huge impact, and the amplitude and frequency of the basic acceleration response will change. In order to analyze the impact of the SSFST system vibration isolator on the track structure response, the foundation is the key test site to test the dynamic impact of the SSFST, and the kurtosis value improved HHT method is used to perform time-frequency analysis and energy analysis on the acceleration response of the track structure.

### 4.1. EMD Analysis

According to the frequency domain analysis in Section 3, it can be seen that the vibration signal has multiple peak points in the frequency domain, and there is a superposition of multiple modal waveforms at the same time. Therefore, we can consider decomposing the vibration signal at the foundation to analyze the vibration waveform of different frequency bands.

According to the characteristics of IMF, for the vibration acceleration at the foundation of SSFST of non-stationary signal, EMD can be used for analysis and processing to obtain the IMF of each frequency band. Through the energy intensity under the characteristic scale reflected by IMF, as shown in the impact intensity of the vibration acceleration signal in each frequency band, it is used to compare the vibration acceleration signal of the foundation under different vibration isolators and fatigue conditions [33].

Since the vibration acceleration signal at the foundation of SSFST presents nonlinear and non-stationary characteristics, it is very suitable to use the EMD method to analyze its modal components. At present, there are few studies in SSFST vibration analysis. Therefore, the use of EMD to obtain the IMF components of the vibration signal at different frequency bands can provide a more comprehensive understanding of the change law of the vibration signal.

**Figure 13:**
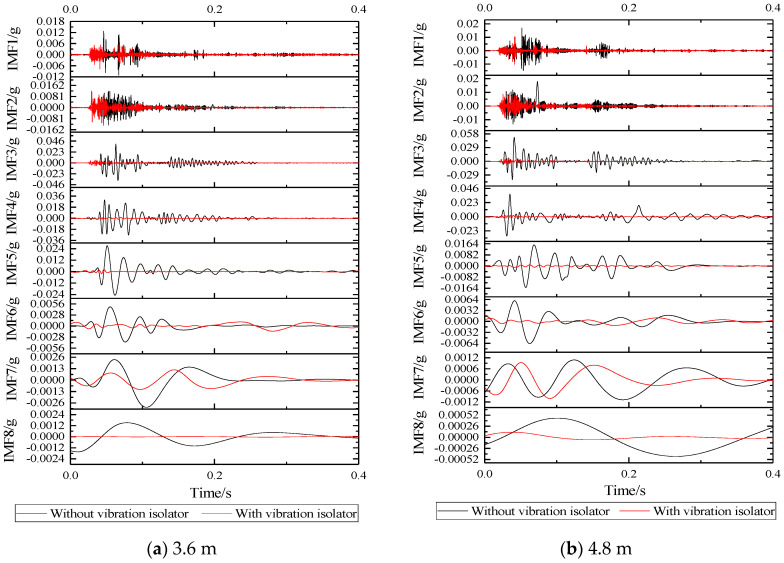
Comparison of basic acceleration EMD waveforms with and without vibration isolators.

**Figure 14:**
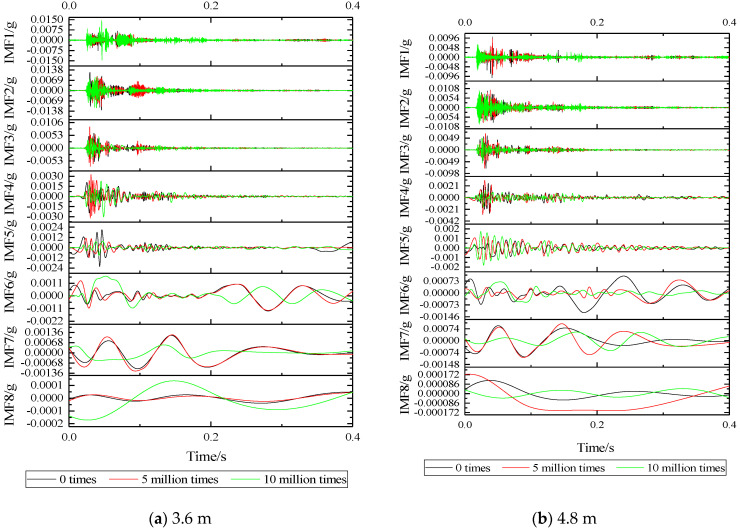
EMD waveform of foundation acceleration under different fatigue load times.

Using the above steps, EMD transformation is performed on the vibration acceleration time-domain waveform in Figure 10 to obtain the basic acceleration EMD waveform with or without vibration isolators and the basic acceleration EMD waveform under different fatigue load times, as shown in Figure 13 and Figure 14. It can be seen that the vibration signal can be decomposed into 8 orders by using the EMD method, and the decomposed vibration signal presents a relatively regular pure oscillation characteristic. As the decomposition order increases, the amplitude of the IMF decreases, the period becomes longer, and the frequency decreases. It can be seen that EMD has better adaptability to non-stationary fundamental vibration acceleration signals.

According to Figure 13: IMF of various orders with or without vibration isolators, it can be seen that the vibration amplitudes of IMF1, IMF2, IMF7, and IMF8 of the two are maintained at an order of magnitude, which is relatively closer, in which it can be seen that the vibration isolator has limited damping effect on the ultra-high-frequency band and the ultra-low-frequency band. For IMF3~IMF6, it can be seen that after the installation of the vibration isolator, the vibration amplitude has been significantly attenuated, especially in the frequency range of IMF4, and the vibration IMF at the foundation of the two sizes of SSFST is basically the same.

According to Figure 14, it can be seen that as the number of fatigue loads increases, the IMF component of the vibration acceleration at the foundation changes to a certain extent. At 5 million times of fatigue, the IMF component at the foundation of the two sizes of SSFST has a small change range, and the IMF changes of each order of vibration of the two sizes are consistent. When the number of fatigue reaches 10 million times, the IMF component changes greatly, especially in the IMF1~IMF2 frequency band; the IMF7~IMF8 frequency band drops significantly; and the IMF3~IMF6 frequency band shows irregular changes.

According to the IMF waveform decomposed by the EMD method, the IMF maximum value under different working conditions is summarized and analyzed, as shown in the Table 3 and Table 4. With the increase of IMF order, the frequency and amplitude of vibration show a decreasing trend; after installing the vibration isolator, the maximum value of the IMF waveform of IMF3~IMF8 decreased significantly; comparing the maximum positive value and the maximum negative value of the IMF waveform, it can be seen that the absolute value of the maximum negative value of the IMF without vibration isolator is generally smaller than the maximum positive value, while the absolute value of the maximum negative value of IMF with vibration isolator is generally greater than the maximum positive value; and with the increase of the number of fatigue loads, the maximum value of IMF gradually showed a downward trend.

### 4.2. Kurtosis Analysis

In order to analyze the vibration severity of each IMF in Section 4.1, the kurtosis value is used for research. The kurtosis value is also called the fourth-order central moment, which is often used for statistical estimation of the signal. It is used to express the convexity of the peak of the analyzed vibration signal, has no unit, and is a dimensionless value [34]. The larger the kurtosis value, the sharper the peak of the vibration signal, which means that the vibration at the foundation is more intense in this study. The mathematical expression of the kurtosis value is:


(3) $$K=\frac{E{\left(x-\mu \right)}^{4}}{\sigma^{4}}=\frac{1}{N}\sum_{i=1}^{N}[\frac{x_{i}-\mu }{\sigma }{]}^{4}$$

In the formula, *K*—the kurtosis value of the vibration signal at the foundation; *N*—the number of sampling points in the vibration signal; *μ*—the mean value of the vibration signal; and *σ*—the standard deviation of the vibration signal.

In the field of mechanical engineering diagnosis, it is generally considered that when the kurtosis value is equal to 3, it is the normal kurtosis value, also called zero kurtosis. At this time, the concavity and convexity at the peak of the analyzed waveform signal is consistent with the normal distribution waveform. When σ decreases, it indicates that the vibration signal is more concentrated, and the kurtosis value increases. On the contrary, when σ increases, this indicates that the peak value of the vibration signal is more dispersed, and the kurtosis value decreases. According to the above rule, when the kurtosis value is greater than 3, it is recorded as positive, and when the kurtosis value is less than 3, it is recorded as negative. By using the kurtosis index to analyze the dramatic magnitude of IMF changes, it can also be used to understand the degree of peak fundamental vibration acceleration in each frequency band. Therefore, the IMF kurtosis values of each order in Section 4.1 are calculated, as shown in Table 5.

**Figure 15:**
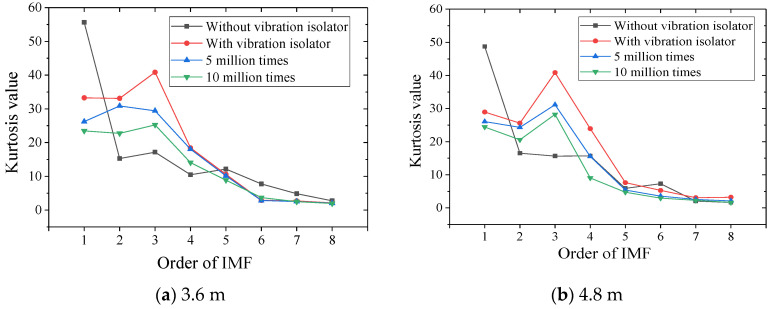
IMF kurtosis trend graph of basic acceleration.

Drawing the kurtosis values of each order into the change waveform shown in Figure 15, we can see that:

(1) When there is no vibration isolator, the IMF1 kurtosis value of the vibration signal at the foundation is larger, and the kurtosis value decreases sharply at IMF2 and is smaller than the working condition of the vibration isolator. After IMF3, the kurtosis value changes steadily, gradually decreases, and gradually becomes negative kurtosis at IMF7~IMF8;
(2) After installing the vibration isolator, the IMF kurtosis values of each order are reduced, the IMF2~IMF4 is higher than without vibration isolators, and the peak signal density of the IMF3 frequency band is the highest. As the number of fatigue increases, the kurtosis value of this frequency range first increases and then decreases, indicating that the fatigue load will affect the distribution of the vibration acceleration IMF peak point at the foundation.

In order to analyze the variation range of the kurtosis value under different installation conditions of vibration isolators and the number of fatigue loads, the kurtosis value change ratios after installing the vibration isolator, after 5 million times of fatigue and after 10 million times of fatigue are summarized, as shown in the Table 6. It can be seen that IMF2~IMF4 of 3.6 m SSFST ground vibration has a relatively large increase after installing the vibration isolator, while IMF2~IMF4 and IMF7~IMF8 of 4.8 m SSFST ground vibration have a significant increase; under the action of fatigue load, all orders of IMF showed a downward trend. The decrease range of IMF5~IMF8 of 3.6 m SSFST ground vibration was greater than 4.8 m SSFST, while the reduction ratio of IMF1~IMF4 was less than 4.8 m SSFST.

### 4.3. Hilbert Envelope Spectrum Analysis

In the field of mechanical engineering, Hilbert envelope spectrum is often used for fault diagnosis. This method is based on the traditional spectrum analysis technology, combined with the envelope detection of the vibration signal, which can remove the high-frequency components of the vibration signal and amplify the defect signal to facilitate diagnosis and analysis. The function after the Hilbert transformation can be used to construct the analytic function, and then the Hilbert envelope spectrum can be obtained by performing FFT on this basis [35,36].

The Hilbert envelope spectrum analysis method is as follows.

The original vibration signal undergoes Hilbert transform, which makes the vibration signal produce a phase shift of 90°. The transformed analytical signal can be used to construct an envelope signal. The Hilbert transform is defined as:

(4) $$\hat{x}(t)=H[x(t)]=\int_{-\infty }^{+\infty }\frac{x(\tau )}{\pi (t-\tau )}d\tau =x(t)\cdot \frac{1}{\pi t}$$

$\hat{x}(t)$ is the signal after the original vibration signal x(*t*) is transformed by Hilbert, and then the analytical signal can be obtained:


(5) $$Z(t)=x(t)+j\hat{x}(t)$$

On this basis, the envelope function *A*(*t*) is constructed, which is defined as follows:

(6) $$A(t)=\frac{\hat{x}(t)}{x(t)}$$

where: *A*(*t*)—the envelope signal of the original vibration signal *x*(*t*).

The instantaneous phase *φ*(*t*) is defined as:


(7) $$\varphi (t)=arctan[\frac{\hat{x}(t)}{x(t)}]$$

**Figure 16:**
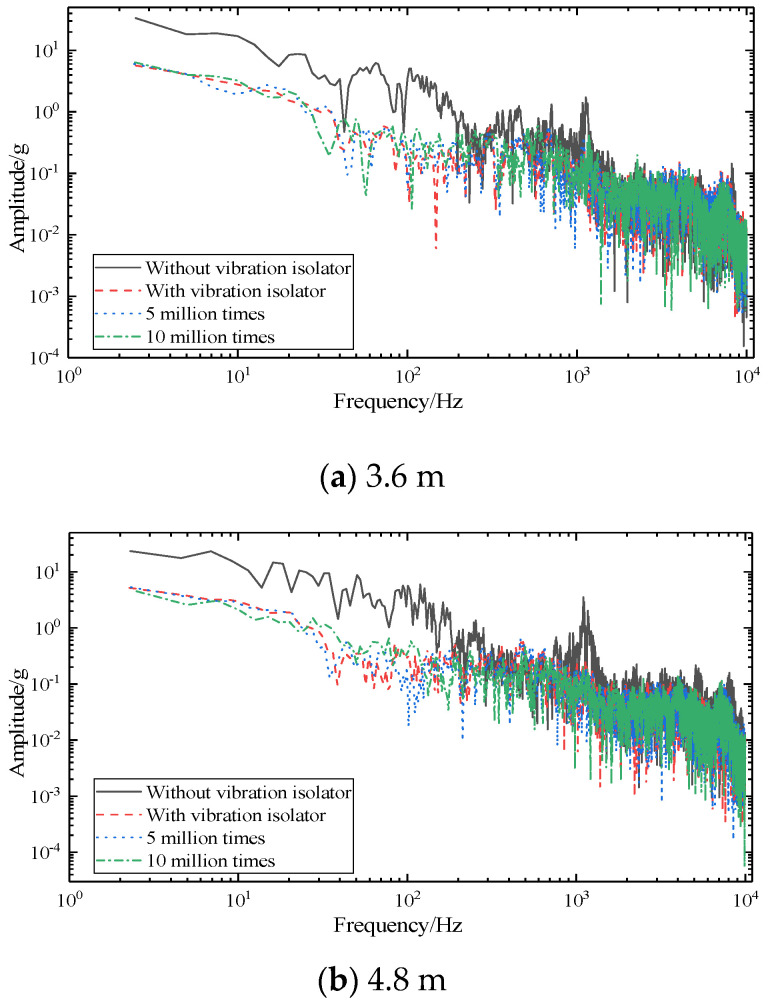
Hilbert envelope spectrum of basic acceleration.

Since the vibration of SSFST foundation is most concerned about the middle and low frequencies, according to the above steps, the original vibration signal is detected and the high-frequency components are removed, so that the Hilbert envelope spectrum is drawn. The low-frequency defect signals in the spectrum are easier to identify through the method. Therefore, the Hilbert envelope spectrum analysis is performed on the SSFST basic vibration acceleration of two lengths, as shown in Figure 16.

It can be seen that after Hilbert detection, the amplitude of frequencies above 1000 Hz is greatly reduced. The vibration acceleration envelope signal when there is no vibration isolator can be regarded as a fault signal. It can be seen that the frequency amplitude below 110 Hz is larger than that of the vibration isolator. After the fatigue load, the Hilbert envelope waveform has certain fluctuations, especially in the frequency range between 10 Hz and 30~50 Hz. The laws reflected under different fatigue loading times are consistent with the previous analysis results, that is, 5 million times of fatigue has a small effect, and 10 million times of fatigue has a greater effect.

According to the Hilbert envelope spectrum, as the frequency increases, the Hilbert amplitude gradually decreases, indicating that the foundation structure receives mainly the drop hammer impact transmitted from the track structure. The maximum Hilbert amplitudes of the 3.6 m SSFST ground vibration under the four working conditions are 33.413 g, 6.408 g, 6.000 g, and 5.670 g, respectively. In the range of 0~80 Hz, the prominent protruding points without vibration isolators are located at 7.50 Hz, 24.98 Hz, and 64.52 Hz, and there is a significant depression point at 42.49 Hz. There are many fluctuations in the basic Hilbert amplitude under different fatigue loading times. In the range of 0~80 Hz, before fatigue, 5 million times of fatigue, and 10 million times of fatigue, the first large fluctuation occurs at about 10 Hz, where the Hilbert amplitudes are 17.061 g, 2.788 g, 1.935 g, and 3.245 g. The maximum Hilbert amplitudes of the 4.8 m SSFST ground vibration under the four working conditions are 23.431 g, 5.316 g, 5.173 g, and 4.490 g, respectively. In the range of 0~80 Hz, the prominent protruding points without vibration isolators are located at 6.89 Hz, 16.08 Hz, 22.98 Hz, 32.16 Hz, and 50.54 Hz, and there is a significant depression point at 39.06 Hz. The first large fluctuation appeared at 5 Hz, and the Hilbert amplitudes were 17.622 g, 3.919 g, 3.731 g, and 2.577 g, respectively.

## 5. Vibration Level Analysis

### 5.1. Frequency Division Vibration Level Analysis

According to the “Technical Specification for Floating Slab Track” (CJJ/T 191-2012) [37], when evaluating the vibration-damping performance of the SSFST in this study, the test frequency range that needs to be used is 1~200 Hz. The vertical acceleration should be used in the test, and the vibration acceleration with or without vibration isolation measures should be tested for comparison by calculating the root mean square difference Δ*La* of the divided frequency vibration level when the SSFST with or without vibration isolator is used as the evaluation parameter. The difference Δ*L_max_* of the frequency division vibration level is used as the evaluation quantity of the SSFST vibration-damping effect, and the calculation formula is as follows:


(8) $$\Delta L_{a}=10\mathrm{lg}(\overset{n}{\underset{i=1}{\sum }}10^{\frac{VL_{q}\left(i\right)}{10}})-10\mathrm{lg}(\overset{n}{\underset{i=1}{\sum }}10^{\frac{VL_{h}\left(i\right)}{10}})$$

(9) $$\Delta L_{\max}=\underset{i=1\to n}{\max}\left[VL_{q}\left(i\right)-VL_{h}\left(i\right)\right]$$

(10) $$\Delta L_{\min}=\underset{i=1\to n}{\min}\left[VL_{q}\left(i\right)-VL_{h}\left(i\right)\right]$$

where *V_Lq(i)_*—in this study, the measured vertical acceleration on the ground without vibration isolators is the divided-frequency vibration level when the vertical acceleration is in the 1/3 octave band;

*V_Lh(i_*_)_—in this study, the tested vertical acceleration is the divided-frequency vibration level when the vertical acceleration is in the 1/3 octave band.

**Figure 17:**
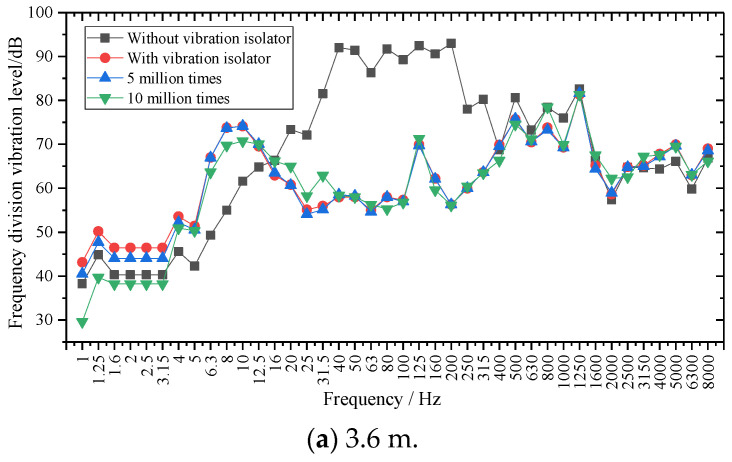

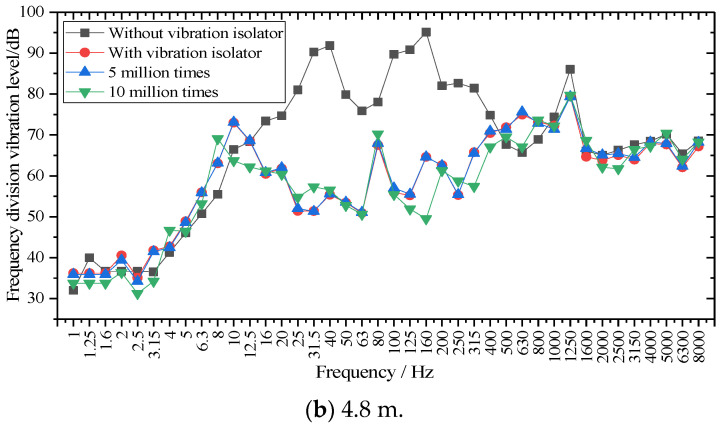
1/3 octave waveform at the base of SSFST.

**Figure 18:**
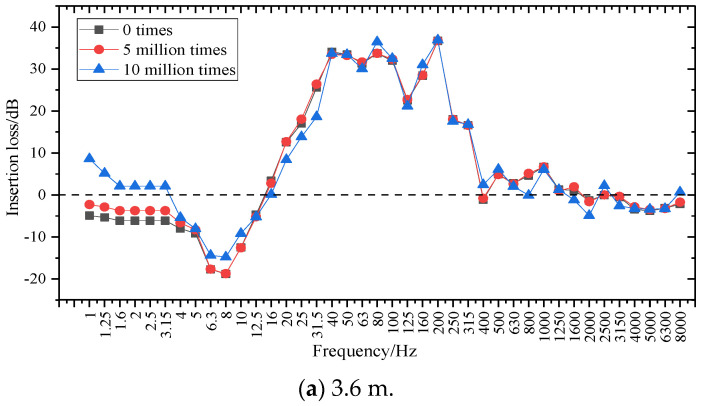

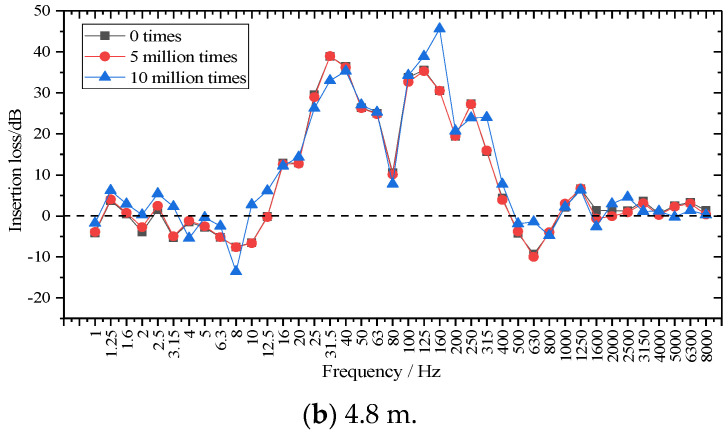
Insertion loss curve at the foundation of SSFST.

The 1/3 octave frequency at the basis of the two lengths of SSFST is shown in Figure 17, and the insertion loss analysis is shown in Figure 18.

According to Figure 17, in the range of 1~200 Hz, the 1/3 octave at the foundation is distributed at 20~95 dB, and the peak value is at 100~200 Hz when the vibration isolator is not installed, located at 40 Hz, 80 Hz, 125 Hz, 200 Hz, 500 Hz, 800 Hz, and 1250 Hz, respectively, and their frequency positions correspond to the seven peak points analyzed in Section 3.2 (44.99 Hz, 89.98 Hz, 132.48 Hz, 194.98 Hz, 534.93 Hz, 789.91 Hz, and 1254.84 Hz) and are consistent. After the vibration isolator is installed, the vibration level at the base is significantly reduced in the frequency range of 16~400 Hz, and the peak value is at 8~12.5 Hz. The peak points correspond to 10.50 Hz, 134.98 Hz, 524.93 Hz, and 1222.35 Hz. The peak point of the vibration level curve under the fatigue load of 5 million times changes little, while the vibration level curve fluctuates greatly after the fatigue load of 10 million times.

From the comparison of Figure 17a,b, it can be seen that with the increase of fatigue load, the 1/3 octave of the foundation at different frequency bands fluctuates to a certain extent, but the variation range is small. The two-length SSFSTs have very similar basic frequency division vibration level curves under various working conditions, and each amplitude frequency division vibration level of the 4.8 m SSFST foundation is slightly smaller than that of the 3.6 m SSFST foundation.

In order to study the effect of fatigue on the insertion loss of steel spring isolators, the fatigue loss of two SSFSTs was plotted as a function of fatigue load, as shown in Figure 18. It can be seen that in the range of 1~200 Hz, the vibration isolation effect of the vibration isolator on the foundation vibration is mainly reflected in the 16~400 Hz range. This phenomenon is consistent with the results in Section 3.2, that is, the vibration damping of the steel spring vibration isolator is mainly reflected in low frequency. At the same time, the insertion loss of the base frequency in the 5~16 Hz range is negative, indicating that the vibration in this frequency range is intensified, which is consistent with the conclusion that the maximum amplitude appears around 10 Hz after the isolator is installed in the analysis results of the amplitude spectrum (RMS). With the increase of fatigue load, the vibration isolator has a certain fluctuation in the insertion loss of the foundation at different frequency bands, but the variation amplitude is limited.

### 5.2. Z Vibration Level Analysis

**Figure 19:**
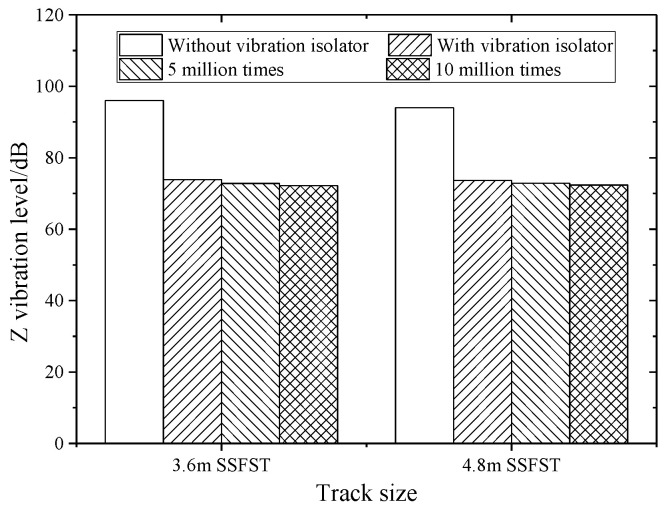
The Z vibration level of the foundation.

The Z vibration levels (VLz) at different track structure components of the two SSFSTs are shown in Table 7 and Figure 19. It can be seen that during the drop hammer impact, when weighing according to ISO2631-1:1985 [38], the spring vibration isolator’s foundation VLz gradually decreases before and after installation, after 5 million fatigue tests, and after 10 million fatigue tests. The Z vibration level results of the two sizes of SSFST are similar, and it can be seen that the increase in fatigue load will not weaken the vibration-damping effect of SSFST. The relevant literature [39] shows that the load caused by the operation of the subway line in one year is equivalent to 2 million fatigue loads. In this experiment, the vibration-damping effect changes little after 5 million and 10 million times, which just proves that the floating slab track can still guarantee a good vibration-damping effect after about 2.5 years and 5 years, which shows that the track structure has high reliability and has a shelf life of vibration-damping effect about 5 years.

### 5.3. Comparison with Cast-in-Place SSFST Railway Field Test

The combined shear hinge prefabricated SSFST has not been widely laid on the railway site at present. In order to reflect the advanced nature of the track slab, we compared the field test data of the cast-in-place SSFST previously tested with it. The results show that this new type of combined shear hinge SSFST is not only beneficial to the rapid construction on site but also has good engineering application value, and its vibration-damping effect can also reach the level of cast-in-place SSFST. At the same time, the frequency response of the vibration isolator reflected by the prefabricated SSFST indoor test and the cast-in-place SSFST field test is consistent, and it can be seen that the drop-weight method has high feasibility for evaluating the vibration damping characteristics of the SSFST that has not been constructed on site.

**Figure 20:**
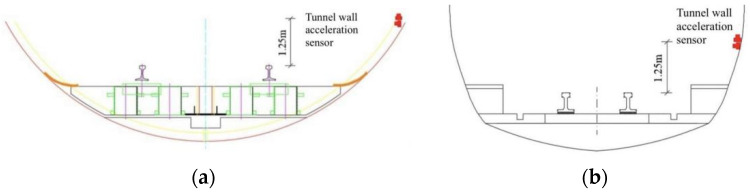
Layout of tunnel wall acceleration test points. (**a**) Cast-in-place SSFST section. (**b**) OMT section.

**Figure 21:**
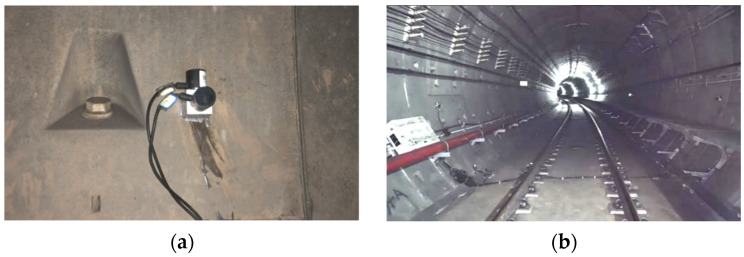
Field test diagram. (**a**) Acceleration measuring point of tunnel wall. (**b**) Overall view.

The wall vibration of a cast-in-place SSFST tunnel of a subway line is selected to be measured by an acceleration sensor, and the length of the cast-in-place SSFST is 25 m. At the same time, an ordinary monolithic track (OMT) section was selected for comparison with the tunnel wall vibration, and each measurement section used an acceleration sensor to measure the vertical acceleration of the tunnel wall (Figure 20). The site layout is shown in the Figure 21.

**Figure 22:**
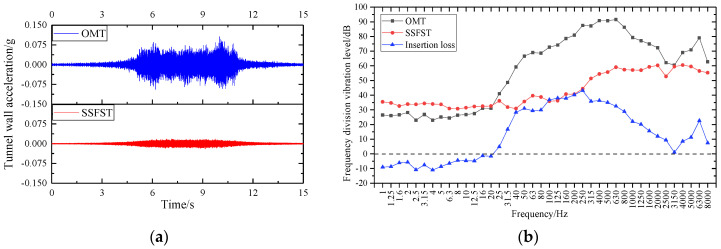
Comparison of vertical vibration of tunnel wall. (**a**) Acceleration time domain curve. (**b**) Frequency division vibration level and insertion loss.

Through the test, the comparison chart of tunnel wall vibration in Figure 22 is obtained. For the tunnel wall acceleration, the SSFST section is significantly smaller than the OMT section, and the amplitude is about 0.2~0.3 of the OMT section. It can be seen that SSFST has excellent vibration isolation effect, which can filter and weaken the vibration very efficiently, which is of great significance to ensure the vibration damping requirements along the subway line. In the frequency band below 12.5~16 Hz, the insertion loss of the frequency division vibration level of SSFST is negative, indicating that the setting of SSFST will aggravate the vibration response of this frequency band. The reason is that the natural vibration frequency of SSFST is mainly located in this section. Excitation in the frequency band results in increased vibration in this frequency band. The insertion loss curve of the frequency band near 3150 Hz shows a valley value, and the vibration-damping effect of the high-frequency band also decreases, indicating that the vibration-damping effect of SSFST is mainly concentrated in the 16~3150 Hz frequency band. Through the analysis of the insertion loss curve, it can be seen that the vibration-damping effect reaches its peak value near 200~250 Hz, which indicates that the vibration isolation and filtering of the steel spring vibration isolator for medium and high-frequency signals are mainly concentrated in this frequency band. Therefore, the vibration damping characteristics of SSFST reflected by the field test are consistent with the results of the laboratory test.

According to “Technical Guidelines for Environmental Impact Assessment” HJ453-2008 [40], for the environmental vibration caused by train operation, the train speed correction formula at different speeds is:

(11) $$C_{v}=20\mathrm{lg}\frac{v}{v_{0}}$$

where:

*v*_0_—the reference speed of the source intensity, in km/h;
*v*—the speed of the train, in km/h.

Taking the data measured when 20 trains pass through the test section, the VL_Z_ value and the vibration difference value ΔVL_Z_ of the tunnel wall measurement point of each test section under the two track bed forms are analyzed and statistically obtained, see Table 8. After the vehicle speed is corrected to the same speed, the Z vibration level of the tunnel wall in the SSFST section is significantly smaller than that in the OMT section, and the insertion loss ΔVL_Z_ is 24.42 dB. Compared with the combined shear hinge prefabricated SSFST in this test, the vibration-damping effect of the two types of SSFST is above 20 dB. Due to the different working conditions without vibration isolators, the vibration level and the value of the vibration-damping effect are different, but the overall law and the magnitude of the vibration-damping effect are consistent and the reliability is high.

In summary, the prefabricated SSFST indoor test and the cast-in-place SSFST field test reflect the same vibration damping frequency response law of the isolator. It can be seen that the drop-weight method has high feasibility for evaluating the vibration damping characteristics of the SSFST that is not constructed on site.

## 6. Conclusions

By establishing a full-scale model of the combined shear hinge SSFST, the drop hammer impact test of the combined shear hinge SSFST under different isolator assembly conditions and fatigue times was carried out. The dynamic response of the foundation (ground) of two lengths of SSFST structures under the impact of a drop weight was investigated. According to the time domain, frequency domain, vibration level, and other indicators, the vibration-damping performance of the combined shear hinge SSFST structure is compared, and the vibration excitation response of the SSFST foundation under different working conditions is evaluated. The main conclusions are as follows:

(1) Through amplitude (RMS) analysis, EMD analysis, kurtosis value analysis, Hilbert envelope spectrum analysis, and vibration level analysis, it can be seen that this novel type of combined shear hinge SSFST can have excellent isolation effect on vibration and can still maintain good vibration damping ability within 10 million fatigue loads (about 5 years).
(2) By comparing the magnitude (RMS) spectrum, the IMF waveform of the EMD method, the frequency division vibration level curve and the insertion loss curve, it can be seen that the frequency of the peak point of the frequency division vibration level curve corresponds to the peak point frequency of the amplitude (RMS) spectrum. The vibration damping results of each frequency band reflected by the insertion loss curve are consistent with the IMF display results after EMD transformation, that is, the vibration-damping effect of the steel spring vibration isolator mainly acts on the middle and low-frequency bands of 16–400 Hz.
(3) According to the amplitude (RMS) spectrum, frequency division vibration level curve, and insertion loss curve, it can be seen that the vibration near 10 Hz will be intensified after the vibration isolator is installed, while the human body is more sensitive to the vibration response in the frequency range of 0~80 Hz. Therefore, this problem should be improved in the subsequent SSFST design to weaken the vibration response of the floating slab to the foundation in this frequency band.
(4) The vibration indicators and variation laws of the two sizes of SSFST are similar, and the acceleration amplitude and attenuation laws are consistent. The vibration response of 4.8 m SSFST is slightly smaller than that of 3.6 m SSFST. Since 3.6 m SSFST is easier to lay a smooth line, in practical applications, 3.6 m SSFST should be arranged in the curved section, and 4.8 m SSFST should be arranged in the straight-line section with higher train speed.
(5) Compared with the field experiment method, the drop-weight method can also systematically study the vibration characteristics of this novel combined shear hinge SSFST before laying, and the displayed SSFST vibration damping characteristics are consistent with the field test and can conduct a comprehensive evaluation of the vibration-damping effect of SSFST at a lower cost.

## Figures and Tables

**Table 1:** SSFST test component parameters.

Length	Component	Amount	Parameter
3.6 m	Prefabricated slab	1	3600 mm(length) × 2700 mm(width) × 340 mm(thickness), C50
	Vibration isolator	6	6.66 kN/mm
	Shear hinge	2	SDT-55 × 870
4.8 m	Prefabricated slab	1	4800 mm(length) × 2700 mm(width) × 340 mm(thickness), C50
	Vibration isolator	6	7.50 kN/mm
	Shear hinge	2	SDT-55 × 870

**Table 2:** Time domain analysis comparison of vibration acceleration (unit: g).

Fatigue Load	Without Vibration Isolator (WOVI)		With Vibration Isolator (WVI)		5 Million Times (5 MT)		10 Million Times (10 MT)	
Type	3.6 m	4.8 m	3.6 m	4.8 m	3.6 m	4.8 m	3.6 m	4.8 m
Max	0.0657	0.0563	0.0162	0.0143	0.0154	0.0141	0.0138	0.0127
Minimum	−0.0660	−0.0627	−0.0165	−0.0145	−0.0160	−0.0143	−0.0153	−0.0139
Peak	−0.0660	−0.0627	−0.0165	−0.0145	−0.0160	−0.0143	−0.0155	−0.0139
Peak-to-peak	0.1317	0.1190	0.0327	0.0284	0.0314	0.0280	0.0291	0.0226
Effective value	0.0105	0.0099	0.0021	0.0018	0.0020	0.0017	0.0018	0.0016

**Table 3:** Summary of the maximum negative values of each IMF waveform (unit: g).

Size	Working Condition	IMF1	IMF2	IMF3	IMF4	IMF5	IMF6	IMF7	IMF8
3.6 m	Without vibration isolator	−0.0112	−0.0128	−0.0370	−0.0271	−0.0248	−0.0041	−0.0031	−0.0016
	With vibration isolator	−0.0077	−0.0131	−0.0062	−0.0025	−0.0022	−0.0013	−0.0011	0.0000
	5 million times	−0.0061	−0.0108	−0.0076	−0.0032	−0.0012	−0.0013	−0.0012	0.0000
	10 million times	−0.0145	−0.0116	−0.0044	−0.0030	−0.0009	−0.0011	−0.0005	−0.0002
4.8 m	Without vibration isolator	−0.0149	−0.0132	−0.0385	−0.0314	−0.0159	−0.0065	−0.0011	−0.0004
	With vibration isolator	−0.0103	−0.0084	−0.0076	−0.0028	−0.0009	−0.0012	−0.0010	−0.0001
	5 million times	−0.0089	−0.0086	−0.0077	−0.0030	−0.0012	−0.0006	−0.0010	−0.0002
	10 million times	−0.0066	−0.0093	−0.0055	−0.0017	−0.0018	−0.0006	−0.0006	0.0000

**Table 4:** Summary of the maximum positive value of each IMF waveform (unit: g).

Size	Working Condition	IMF1	IMF2	IMF3	IMF4	IMF5	IMF6	IMF7	IMF8
3.6 m	Without vibration isolator	0.0127	0.0122	0.0398	0.0297	0.0270	0.0048	0.0023	0.0015
	With vibration isolator	0.0066	0.0121	0.0061	0.0021	0.0021	0.0010	0.0012	0.0000
	5 million times	0.0051	0.0096	0.0088	0.0033	0.0011	0.0013	0.0012	0.0000
	10 million times	0.0142	0.0089	0.0047	0.0023	0.0008	0.0017	0.0005	0.0001
4.8 m	Without vibration isolator	0.0169	0.0180	0.0507	0.0366	0.0155	0.0060	0.0011	0.0005
	With vibration isolator	0.0103	0.0094	0.0058	0.0026	0.0008	0.0010	0.0009	0.0001
	5 million times	0.0093	0.0100	0.0068	0.0031	0.0012	0.0008	0.0011	0.0002
	10 million times	0.0064	0.0096	0.0049	0.0013	0.0017	0.0007	0.0005	0.0000

**Table 5:** IMF kurtosis value of vibration acceleration of SSFST system components.

Size	Working Condition	IMF1	IMF2	IMF3	IMF4	IMF5	IMF6	IMF7	IMF8
3.6 m	Without vibration isolator	55.651	15.281	17.178	10.485	12.149	7.734	4.863	2.739
	With vibration isolator	33.294	33.107	40.849	18.419	10.579	2.906	2.704	2.116
	5 million times	26.238	30.885	29.416	18.065	10.149	2.851	2.531	2.063
	10 million times	23.472	22.742	25.266	14.071	8.862	3.725	2.484	1.967
4.8 m	Without vibration isolator	48.728	16.509	15.663	15.672	5.911	7.301	2.058	1.633
	With vibration isolator	28.936	25.593	40.848	23.911	7.629	5.288	3.099	3.227
	5 million times	26.062	24.315	31.146	15.54	5.404	3.571	2.549	2.13
	10 million times	24.424	20.564	28.221	9.048	4.728	2.949	2.288	1.677

**Table 6:** The ratio of changes in kurtosis values.

Size	Working Condition	IMF1	IMF2	IMF3	IMF4	IMF5	IMF6	IMF7	IMF8
3.6 m	With vibration isolator	0.598	2.167	2.378	1.757	0.871	0.376	0.556	0.773
	5 million times	0.788	0.933	0.720	0.981	0.959	0.981	0.936	0.975
	10 million times	0.705	0.687	0.619	0.764	0.838	1.282	0.919	0.930
4.8 m	With vibration isolator	0.594	1.550	2.608	1.526	1.291	0.724	1.506	1.976
	5 million times	0.901	0.950	0.762	0.650	0.708	0.675	0.823	0.660
	10 million times	0.844	0.804	0.691	0.378	0.620	0.558	0.738	0.520

**Table 7:** The Z vibration level of the foundation weighting (VLz, unit: dB).

Track Size	Data	Track Structure Components			
		Without Vibration Isolator	With Vibration Isolator	After 5 Million Fatigue	After 10 Million Fatigue
3.6 m	Z vibration level	96.0	73.9	72.8	72.2
	Insertion loss		22.1	23.2	23.8
4.8 m	Z vibration level	94.0	73.7	72.9	72.4
	Insertion loss		20.3	21.1	21.6

**Table 8:** Tunnel wall VL_Z_ and its vibration difference ΔVL_Z_.

Track Bed Form	OMT	SSFST
VL_Z_ (dB)	70.83	46.61
Speed (km/h)	71.36	73.00
Corrected value after the speed is unified (dB)	0.00	−0.20
VL_Z_ after speed correction (dB)	70.83	46.41
Insertion loss ΔVL_Z_ (dB)	24.42	

## Data Availability

Not applicable.
